# Preparedness of Medical Graduates to serve in clinical settings independently: An exploratory qualitative study

**DOI:** 10.12669/pjms.38.4.5517

**Published:** 2022

**Authors:** Zafar Ali Choudry, Ayesha Ayub, Sumera Ehsan Badar

**Affiliations:** 1 Prof. Dr. Zafar Ali Choudry, FCPS, FRCS. Vice Chancellor, Faisalabad Medical University, Faisalabad, Pakistan; 2 Dr. Ayesha Ayub, MBBS, MME/MHPE. Demonstrator, HPERD Department, Faisalabad Medical University, Faisalabad, Pakistan; 3 Dr. Sumera Ehsan Badar, MBBS, M.Phil., MME, Assistant Professor and HOD HPERD Department, Faisalabad Medical University, Faisalabad, Pakistan

**Keywords:** House officers, Readiness, Graduate students, Skills

## Abstract

**Objectives:**

To assess the preparedness of fresh medical graduates to perform the duties of an effective house officer in clinical settings independently.

**Methods:**

A qualitative exploratory descriptive study was conducted at a public sector tertiary care teaching hospital from September to October, 2021. A total of 14 interviews of the serving house officers were conducted (7 were from Medicine and Allied and seven were from Surgical and Allied). A verbatim Thematic analysis was done.

**Results:**

Initial analysis revealed 45 codes which were ultimately reduced to five main themes namely **1.** Transition from studentship to house officers with sub-themes (**1a)** Sense of responsibility, **(1b)** Hectic and long duty hours**, (1c)** Proper orientation and guidance, **2**. Deficient skill Training during educational journey with, **(2a)** Deficiency of practical and applied aspects, **(2b)** inconsistent and varying training patterns, **(2c)** self-perception and evaluation of preparedness**, 3**. Lack of awareness about Hospital settings and working system with sub-themes **(3a)** Support from other doctors, **(3b)** Being recognized as a doctor in hospital**, 4**. Inter-professional co-ordination gaps having sub-themes **(4a)** Communication gap, **(4b)** Mutual respect as a team and **5**. Impact of COVID-19 with sub-theme **(5a)** Online teaching with no interaction and **(5b)** segue and progressive skill training.

**Conclusion:**

Medical graduates are not confident and well prepared to take the responsibility of patient care independently in clinical settings. Reforms in undergraduate curricula regarding skill training, hospital setup and workings and inter-professional education are advocated by young doctors to enhance their competencies for professional life.

## INTRODUCTION

Great emphasis is now being paid to develop undergraduate medical curricula in a way that will prepare the students who are skillful enough to practice independently after their graduation.^1^ Newly graduated students are usually not well prepared to deal with acute conditions and face difficulty in their initial transition phase.^2^ The transition from the role of a student to that of a practicing doctor responsible for providing health care is very stressful and challenging.^3^ Undergraduate curricular content and training provided during educational journey is directly associated with the preparedness of students to be able to practically and successfully involved in the clinical settings.^4^ Various studies have shown that graduated students feel uncomfortable to act as health professionals independently and find it difficult to believe in themselves during transition phase.^5^ Lack of preparedness is a result of inadequate training and exposure to actual clinical conditions during educational journey.^6^ Fresh graduates find it challenging to take the responsibility of an independent health care provider and takes time to adjust in their new identity of being a doctor from being a student.^7^ Increased level of anxiety and more burnout is reported in young doctors which is directly associated with the pressure faced by them to perform in the clinical settings independently and to take the responsibility of patient management on their own.^8^ All around the globe, it is emphasized to reform the medical curriculum in a way to incorporate the skills and attitude along with the knowledge necessary to practice medicine when the students change their role from being a learner to that of an independent health care provider.^3^ To give the junior doctors confidence and reduce the stress associated with the transition phase, different programmes had been introduced at undergraduate level and in the initial period of internship to make them well prepared for the role of a good practicing doctor.^2^

During literature search, it was observed that in south-Asian region the graduates have failed to serve the patients independently and are not well prepared for their transition.^9^ Although the importance has been established about the role of fresh graduates in providing care to patients, a gap was found in south Asia the preparedness of graduate students and their experience about the transition phase from being a student to an independent practicing doctor. This study was designed to the preparedness of newly graduated students in Pakistan to act as house officers independently and the problems faced by them during the transition phase. The objective was to assess the preparedness of fresh graduates to perform the duties as doctors in clinical settings independently.

## METHODS

A qualitative exploratory descriptive study was conducted at Allied Hospital Faisalabad, affiliated with the University of Faisalabad from September 2021 to October 2021 after taking permission from ERC committee of FMU No# 48.ERC/FMU/2020-2021/161. After Literature search five questions were initially formulated to check the preparedness level of house officers regarding different roles expected from them including patient management, team participation, task performance and communication. The questions were sent to five medical educationists in different cities of Pakistan and after their feedback three more questions were added regarding practical skills and impact of COVID-19 on their performance. Fourteen House officers from different departments were included in the study who had graduated in last academic year and had started their house jobs within last three months. Interview were recorded after consent and instead of names each participant was given a code. Interviews were transcribed, written and written documents were sent back to participants for their approval. After participants’ approval thematic narrative analysis was done with the consensus of all the researchers to avoid personal bias. Themes and subthemes were generated by open, axial and selective coding of data.

## RESULTS

**Fig.1:**
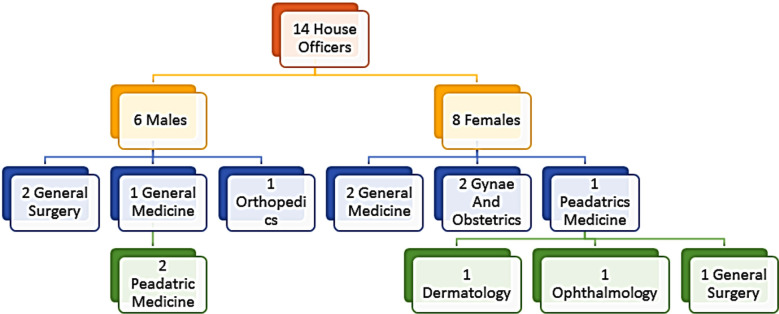
Demographic details of participants.

The demographic details of participants are given in - Fig.1. After analyzing the data five themes were generated **1.** Transition from studentship to house officers with sub-themes (**1a)** Sense of responsibility, **(1b)** Hectic and long duty hours**, (1c)** Proper orientation and guidance, **2**. Deficient skill Training during educational journey with, **(2a)** Deficiency of practical and applied aspects, **(2b)** inconsistent and varying training pattern, **(2c)** self-perception and evaluation of preparedness**, 3**. Lack of awareness about Hospital settings and working system with sub-themes**(3a)** Support from other doctors, **(3b)** Being recognized as a doctor in hospital**, 4**. Inter-professional co-ordination gaps having sub-themes **(4a)** Communication gap, **(4b)** Mutual respect as a team and **5**. Impact of COVID-19 with sub-theme **(5a)** Online teaching with no interaction and **(5b)** segue and progressive skill training (Table-I).

**Table-I:** Themes and Sub-themes finalized after analysis.

Sr no.	Theme	Sub-theme	Description
1.	Transition from student ship to house officers.	**(1a)** Sense of responsibility	*“Now we are responsible for their health”*
		**(1b)** Long and hectic duty hours.	“*I literally had cramps in first week*.”
		**(1c)** Proper orientation and guidance	*“we keep on asking from every one that what we have to do”*
2.	Lack of Training during educational journey	**(2a)** Deficiency of practical and applied aspects.	“*I had knowledge… but no practical experience….in actual situation it matters”*
		**(2b)** inconsistent and varying training patterns.	“*It is Luck” , “those who want to learn, they were being taught, not everyone”*
		**(2c)** self-perception and evaluation of preparedness	Knowledge =60-90%, Skill=0-50% Attitude= 40-70%
3.	Lack of Awareness about Hospital settings and working system	**(3a)** Support from other doctors,	.”*They humiliate us in front of patients, don’t think of us capable enough to be a part of discussion and when we go to that patient again, patients don’t give due respect to us and called us Chota doctor*
		**(3b)** Being recognized as a doctor in hospital	*“They don’t listen to us when they come to know that we are house officers”* **.**
4.	Inter-professional co-ordination	**(4a)** Communication gap,	*“I think initially there was a fear of being unknown”*
		**(4b)** Mutual respect as a team	*“we must work as a team and give them respect, students must be taught about it”*
5	Impact of COVID-19	**(5a)** Online teaching with no interaction and	“*final year was ZOOM year, we are basically landed in the house job directly”*
		**(5b)** segue and progressive skill training	*“if we were involved from 3^rd^ year, I would say conditions will be 50-60% better”*

### 1. Transition from studentship to house officers:

Most of the participants stated that they have a sense of achievement when they started their journey as a house officer but they have doubts about their ability to work independently in the initial 2-3 weeks. “***Emotionally challenging*”, “confused”, “not *confident*” “*doubtful*”** and “***Shaky*”** were the terms used by participants about their initial feelings. ***(1a) Sense of responsibility;*** All the participants felt a sense of responsibility towards the patient care when they started their house job which was missing in their student life. This was associated with a feeling that they are not competent enough to deal with them and to take this responsibility of patient care independently. ***(1b) Hectic and long duty hours:*** Eight out of fourteen participant felt a change in their routines and found it difficult to work for long hours. The participants usually felt tired and exhausted due to long duty hours and felt that it leads to decreased work efficacy. ***(1c) Proper orientation and guidance:*** The new house officers also felt that they are just pushed into wards and no orientation about their role as a house officer is given to them. They were totally unaware of their duties and what is being expected from them. This was an additional factor which leads to anxiety and stress while working in hospital.

### 2. Deficient skill Training during educational journey:

All the participants highlighted that in their student life, emphasis was on knowledge component and skill training was not given much weightage in teaching as well as in their assessment. ***(2a) Deficiency of practical and applied aspects;*** Twelve out of fourteen participants said that during their student life, they were more interested in theoretical component, practical and applied aspects were not as such a part of their training and assessment so they didn’t give much importance to it and paid more importance to theoretical component due to the assessment strategy of medical institute. Most of the participants highlighted that they didn’t Know how to apply sutures, how to get prepared for surgery, how to take samples in different age groups and give intra venous injections when they started their house jobs. ***(2b) Inconsistent and varying training patterns:*** Another important point highlighted by the participants was lack of uniform training during ward rotations in educational period. There was no structured uniform policy of making students learn when they come to wards and involving them in practical work especially in 3^rd^ year and 4^th^ year. **(2c)** ***Self-perception and evaluation of preparedness:*** When asked to give themselves score from one to 100 on their knowledge skill and attitude when they became house officers, knowledge was rated between 60-90%, skill 0-50% and attitude 40-70% by the participants. Procedural Skills were the main area in which all the participants faced difficulty followed by handling deaths and resolving conflicts which comes under affective domain.

### 3. Lack of awareness about Hospital settings and working system:

All the female participants said that they were not aware of the hospital logistics and faced difficulty locating different areas like Blood bank, registration counters, Laboratories etc. during their duty hours. Most of the male participants said that they knew about hospital logistics but they were not completely aware about the working system initially. ***(3a) Support from other doctors:*** All the participants found that senior post-graduate trainees had a supportive role and had helped them a lot. Six out of fourteen participants indicated that the behavior of registrars and professors was not good and they don’t consider house officers as a part of team. This attitude had a negative impact on patients about the competency of house officers ***(3b) Being recognized as a doctor in hospital:*** Participants said that when they go to other wards for some duty purpose they were not given much importance and house officers were taken for granted.

### 4. Inter-professional co-ordination gaps:

All the participants were aware of the role of paramedics and nurses in a health care team and they appreciated them ***(4a) Communication gap;*** Although the participants were aware of the importance of paramedics and nursing staff, ten out of fourteen faced difficulties in communicating with them initially as they had never worked with them in their student life. All the participants strongly advocated to include inter-professional education at undergraduate level and emphasized the importance of co-ordination between different health professionals. ***(4b) Mutual respect as a team;*** six participants indicated that prior to their house job they thought that doctors are superior then other health professionals but after working with them now their perception was changed and they think that they have equal importance in patient management. Nine participants said they never thought about other health professionals during their student life but after coming to practical life they came to know that a health care team is nothing without them.

### 5. Impact of COVID-19:

All the participants said that COVID-19 pandemic was a major factor due to which they were not well prepared to do their duties independently and were not confident about their skills. ***(5a) Online teaching with no interaction;*** All the participants said that due to online classes they had no practical experience in their final year and this was a reason why they don’t have enough practical skills when they started doing house job. ***(5b) Lack of segue and progressive Skill training;*** Although the participants pointed out COVID-19 as a major factor for their lack of practical skills, they also highlighted that all the skill component is left for final year and in 3^rd^ year and 4^th^ year, neither the faculty nor the students pays much importance to wards. The professional training should be progressive and students should be involved in the clinical settings under supervision from early years to train them well and prepare them to act as doctors after graduation independently and with confidence.

## DISCUSSION

The journey of a medical student consists of a series of steps which help them to acquire knowledge, practical skills and appropriate professional behavior required to fulfil the healthcare needs of the community in a professional way. To take the role of a doctor in a clinical setting after graduation and own the responsibility of patient care is considered a major transition in the life of a medical student and is associated with anxiety and stress of the practical life.^10^ Intern doctors from Australia reported the period of transition as *physically, mentally and emotionally challenging* for them and faced difficulties in managing patients in their initial period.^8^ A study conducted in UK on young doctors about their experience of transition also showed stress and fear of taking the responsibility of patient care independently.^3^ The participants of our study showed similar feelings of taking the patient care responsibility on their own and felt the pressure of managing the patients independently when they started working in hospital after their graduation.

Long and hectic working hours were an additional source of stress for our participants and to adjust to new routines took some time during which their working capacity was reduced. Adjusting to the new schedules and long duty hours were reported by young doctors in UK and Kenya as well and had a slowing effect on their professional activities.^3^,^6^ Long working hours and fatigue had a negative impact on the working doctors and leads to compromised patient care.^11^,^12^ Participants of our study advocated the need of reforming medical curricula to prepare the young doctors for coming professional life in their educational life. This idea is accepted globally to reduce the rate of depression and anxiety among medical students and young doctors.^13^

Inadequate training during undergraduate study years is directly associated with the feeling of incompetence to perform independently during professional life and is a major factor of causing stress and anxiety in young doctors.^3^ On hands training during students life had shown to increase the professional competency of those students and helped them in performing duties in their professional life with ease and they were more confident while performing the tasks and dealing with the patients.^14^ Students who had gone through reformed curricula and had experienced problem based and case based learning strategies during their student life are more prepared to take the role of doctor and are more confident in their transition phase as compared to those who had experienced traditional educational environment.^15^ Lack of proper training was indicated as a major factor for feeling not well prepared during the house job by the participants of this study. It is strongly advocated that medical curricula should be reformed to fulfill the educational needs of students in all the three domains of learning so that they are well prepared to step into their professional lives with confidence and without any fear.^9^,^12^

Patient care is a combined effort which not only involves the doctors but includes other health professionals as well and co-ordination between all of them is integral for accurate management.^16^ in Indonesia, it was reported that lack of communication and interaction between health professionals had a negative impact on patient care and to strengthen the primary care facilities inter-professional co-ordination should be improved.^17^ World Health Organization has highlighted inter-professional education and collaboration as an important component for medical curricula at undergraduate and postgraduate levels and many countries like Brazil, Japan and Oman had made it a part of their teaching strategies for better health care community services.^18^-^21^ Inter-professional collaboration was considered a major contributor in patient management by our participants as well and they all identified the need to include it at undergraduate level for better team work and health care provision for community.

COVID-19 pandemic had a drastic effect on economical, psych-social and educational status of all the countries. In UK, the final year students pointed COVID-19 situation to be a major cause for their lack of preparedness about their transition from students to practicing doctors.^22^ In a study conducted on 13 medical institutes, 54.8% students disagree that clinical skills can be taught by e-learning.^23^ In Pakistan, a study conducted on final year medical students showed that 54.9% were concerned about their competency in practical life due to online classes and no ward interaction.^24^ The participants of our study also had the same view and they highlighted the negative impact of COVID-19 and online classes on their skills and preparedness to act as doctors in clinical settings independently.

### Limitation of the study:

This study was conducted on house officers from a single institute. House officers from other institutes, both public and private, should be explored as well for the same question to verify the results and broaden the actual picture.

## CONCLUSION

Medical graduates are not confident and well prepared to take the responsibility of patient care independently in clinical settings. They feel the pressure of being responsible for the health of patients and don’t feel competent enough to handle the patients on their own especially while performing different practical skills. Reforms in undergraduate curricula regarding skill training, hospital setup and workings and inter-professional education are advocated by young doctors to enhance their competencies for professional life. Online learning due to COVID-19 was indicated as a major cause for lack of practical skills by the house officers of batch 2020.

### Implications:

This study results can help curriculum developers and health policy makers to include integrated practical skill training, inter-professional training and system-oriented education as a compulsory part of undergraduate medical education leading to increased competency of graduating medical doctors’ and ultimately to better patient care provided to the community.

### Authors’ Contribution:

**ZAC** conceived, Analyzed and finalize the manuscript.

**AA**, did data collection, transcription, Analysis and manuscript writing.

**SEB** analysis and finalization of manuscript

**AA** takes the responsibility and is accountable for all aspects of the work in ensuring that questions related to the accuracy or integrity of any part of the work are appropriately investigated and resolved.
